# Selective and Specific Inhibition of the *Plasmodium falciparum* Lysyl-tRNA Synthetase by the Fungal Secondary Metabolite Cladosporin

**DOI:** 10.1016/j.chom.2012.04.015

**Published:** 2012-06-14

**Authors:** Dominic Hoepfner, Case W. McNamara, Chek Shik Lim, Christian Studer, Ralph Riedl, Thomas Aust, Susan L. McCormack, David M. Plouffe, Stephan Meister, Sven Schuierer, Uwe Plikat, Nicole Hartmann, Frank Staedtler, Simona Cotesta, Esther K. Schmitt, Frank Petersen, Frantisek Supek, Richard J. Glynne, John A. Tallarico, Jeffrey A. Porter, Mark C. Fishman, Christophe Bodenreider, Thierry T. Diagana, N. Rao Movva, Elizabeth A. Winzeler

**Affiliations:** 1 Novartis Institutes for BioMedical Research, Novartis Pharma AG, Forum 1 Novartis Campus, CH-4056 Basel, Switzerland; 2 Genomics Institute of the Novartis Research Foundation, 10675 John Jay Hopkins Drive, San Diego, CA 92121, USA; 3 Novartis Institute for Tropical Diseases, 10 Biopolis Road, Chromos #05-01, Singapore 138670; 4 The Scripps Research Institute, 10550 North Torrey Pines Road, La Jolla, CA 92037, USA; 5 Novartis Institutes for BioMedical Research Inc., 250 Massachusetts Avenue, Cambridge, MA 02139, USA

## Abstract

With renewed calls for malaria eradication, next-generation antimalarials need be active against drug-resistant parasites and efficacious against both liver- and blood-stage infections. We screened a natural product library to identify inhibitors of *Plasmodium falciparum* blood- and liver-stage proliferation. Cladosporin, a fungal secondary metabolite whose target and mechanism of action are not known for any species, was identified as having potent, nanomolar, antiparasitic activity against both blood and liver stages. Using postgenomic methods, including a yeast deletion strains collection, we show that cladosporin specifically inhibits protein synthesis by directly targeting *P. falciparum* cytosolic lysyl-tRNA synthetase. Further, cladosporin is >100-fold more potent against parasite lysyl-tRNA synthetase relative to the human enzyme, which is conferred by the identity of two amino acids within the enzyme active site. Our data indicate that lysyl-tRNA synthetase is an attractive, druggable, antimalarial target that can be selectively inhibited.

## Graphical abstract

**Graphical Abstract:**

**Figure:**
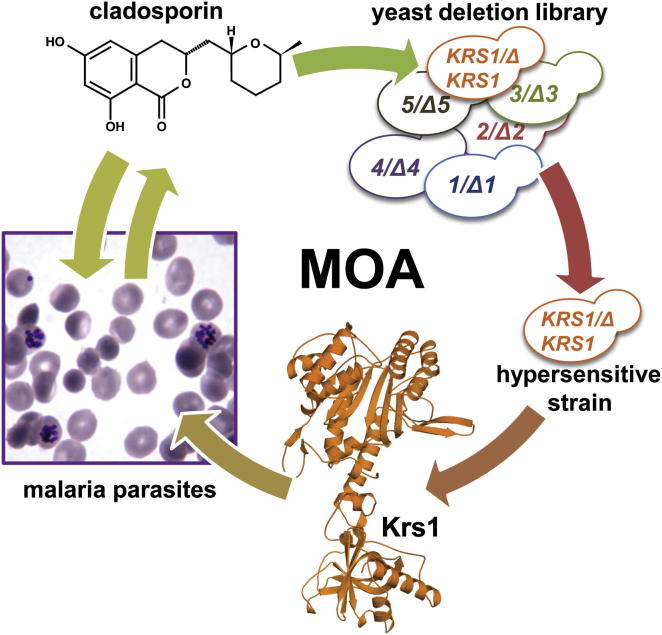

**Highlights:**

► The fungal secondary metabolite Cladosporin inhibits liver- and blood-stage malaria parasites ► Cladosporin specifically targets lysyl-tRNA synthetase (Krs1) ► Cladosporin is >100-fold more potent against parasite Krs1 relative to the human enzyme ► Two amino acids in the Krs1 ATP-binding pocket confer species-selective inhibition

## Introduction

Malaria is a significant health problem, with 225 million annual cases and nearly 3.2 billion people at risk (WHO, 2010). Control and treatment of this disease is compounded by a lack of an effective vaccine. In addition, the emergence of multidrug-resistant parasites has compromised efficacy of many of the frontline chemotherapy treatments. Although there are many effective drugs (Burrows et al., 2011), endoperoxides are the only drug class for which clinically significant resistance has not been reported (Eastman and Fidock, 2009). However, endoperoxides, like many antimalarials, are inactive against the asymptomatic malaria liver stages. To ensure continued malaria control, with an aim for eradication, next-generation antimalarials are required to be active against multidrug-resistant parasites and efficacious against liver- and blood-stage infections.

Traditional drug discovery efforts have revolved around high-value targets identified for their essentiality in the parasite. However, finding targets that are essential for blood and liver malarial stages has been technically challenging, and few inhibitors with these desirable properties are known because of the difficulties associated with simultaneously demonstrating that a target is essential to support viability in both blood and exoerythrocytic stages. An alternative approach to target discovery is to first find compounds with promising activity in phenotypic cell-based screens (Gamo et al., 2010; Guiguemde et al., 2010; Plouffe et al., 2008) and to then determine their mechanism of action through the identification of their specific targets (reviewed in McNamara and Winzeler, 2011). Successful validation of a target from this latter approach provides a proof of concept for small-molecule inhibition and supports continued drug discovery based on rational design of the hit compound.

In order to discover targets for both blood and liver stages, we performed a screen to identify inhibitors of *P. falciparum* blood- and liver-stage proliferation with a natural product library. Cladosporin, a fungal secondary metabolite whose primary target and mechanism of action are not known for any species, was identified as having potent, nanomolar, antiparasitic activity in both blood and liver stages. A member of the isocoumarin class, cladosporin is produced by various fungal genera such as *Cladosporium*, *Aspergillus*, *Eurotium*, and *Chaetomium* (Scott et al., 1971). It has been previously reported to have antifungal (Scott et al., 1971), insecticidal (Grove and Pople, 1981), and antibacterial properties (Anke, 1979) as well as plant growth regulatory effects (Springer et al., 1981) and anti-inflammatory responses in mouse lung tissue (Miller et al., 2010). Using both traditional and systems biology approaches, we show here that cladosporin potently and specifically inhibits cytosolic lysyl-tRNA synthetase in *Plasmodium* spp. In addition, we show that cladosporin is highly selective for the parasite enzyme and that selectivity is in part conferred by the amino acid identity at two key residues in the ATP binding pocket.

## Results

### A Natural Product Screen Identifies Cladosporin with Potent Antiplasmodial Activity

**Figure 1:**
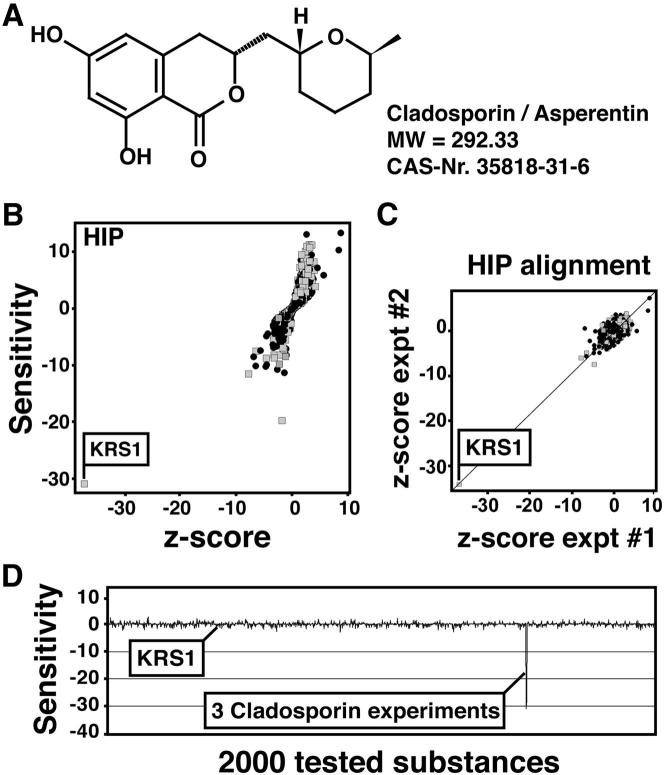
Haploinsufficiency Profiling of Cladosporin Identifies the *KRS1* (A) Chemical structure of cladosporin. (B) HIP analysis of cladosporin at 110 μM. See also Figure S1. Heterozygous strains deleted for essential genes are represented by gray boxes, and strains deleted for nonessential genes are represented by black dots. The deletion strain corresponding to the heterozygous *krs1/KRS1* strain is labeled. (C) Alignment of two additional, independent cladosporin HIP experiments showing reproducibility of the *KRS1* strain hypersensitivity. (D) Cladosporin is the only substance among a diverse collection of 1,800 compounds tested by HIP profiling that significantly affects the heterozygous *KRS1* strain. Sensitivity was calculated as a logarithmic ratio of the relative abundance of any given strain in the treated versus untreated samples and corrected for outliers. The z score couples the sensitivity score to a value proportional to the variation in sensitivity of any HIP strain across the 1,800 diverse compounds tested, thus allowing the identification of nonspecific frequent hitters. For more information, refer to the Supplemental Experimental Procedures.

Small molecules with activity against *P. falciparum* blood-stage parasites were previously identified in a phenotypic screen against a natural product library (Plouffe et al., 2008). Out of the 12,000 natural products, 275 compounds inhibited parasite growth with 50% inhibitory concentration (IC_50_) values in the submicromolar range. These hits were further screened by a high-content image-based assay to determine their ability to block in vitro *P. yoelii* liver-stage development (Meister et al., 2011). Cladosporin (Figure 1A) demonstrated exceptional growth-inhibitory activities against both blood- and liver-stage parasite forms (IC_50_ ∼40–90 nM) while having little effect on the growth or viability of HepG2-CD81 cells (>10 μM) or other human cell lines (Table 1). The high selectivity index of cladosporin against *Plasmodium* parasites compared to mammalian cells (IC_50_/CC_50_ ≥ 111), as well as its equipotent activity against a diverse collection of multidrug-resistant *Plasmodium* lines (Table 1) and cidal action (see Table S1 available online), suggested that further investigation was warranted.

### Chemogenomic Profiling Reveals that Cladosporin Targets Yeast Lysyl-tRNA Synthetase

We next sought to discover the target of cladosporin. Cladosporin demonstrated high micromolar inhibition against the model yeast, *S. cerevisiae*, although activity varied slightly due to compound batch and the growth media conditions (summarized in Table S2). In rich growth media, cladosporin displays a 30% inhibitory concentration (IC_30_) of 110 μM, making it a suitable compound for haploinsufficiency profiling (HIP) assay (Giaever et al., 1999). The HIP target discovery assay is based on a genome-wide collection of heterozygous knockout yeast strains, each of which contains a marked gene deletion (Giaever et al., 2002; Winzeler et al., 1999). It has been shown that heterozygous diploid strains that bear a deletion in one copy of a small molecule's target gene show increased sensitivity to that small molecule relative to those strains that have two copies of the gene (Giaever et al., 1999). This is a likely consequence of decreased expression of the target in the heterozygous diploid strain. Each of these strains also carries a distinct 20 base pair DNA barcode that can be PCR amplified and quantified in competitive growth assays using microarrays containing the barcode complements. As each barcode is linked to a specific strain, barcodes that disappear over time in the presence of a small molecule reveal the identity of the molecular target of the small molecule.

Three independent HIP assays reproducibly identified the heterozygous lysyl-tRNA synthetase (*krs1*/*KRS1*) knockout strain as being the only strain in the collection of 5,803 total strains (covering ∼95% of the genome) that was hypersensitive to cladosporin (Figures 1B and 1C). Because strains that bear deletions in drug pumps or other proteins that attenuate the action of small molecules often also show haploinsufficiency, we also assessed whether the *krs1*/*KRS1* strain is ubiquitously sensitive. Historical profiling of 1,800 compounds (our unpublished data) revealed that the *krs1/KRS1* strain was exclusively sensitive to cladosporin (Figure 1D), indicating a high degree of selectivity for cladosporin. *KRS1* is a nonredundant and essential gene in yeast and functions to load lysine onto the corresponding tRNA molecule for protein translation (Mirande and Waller, 1988).

### Overexpression of *KRS1* Decreases Cladosporin Antifungal Activity

**Figure 2:**
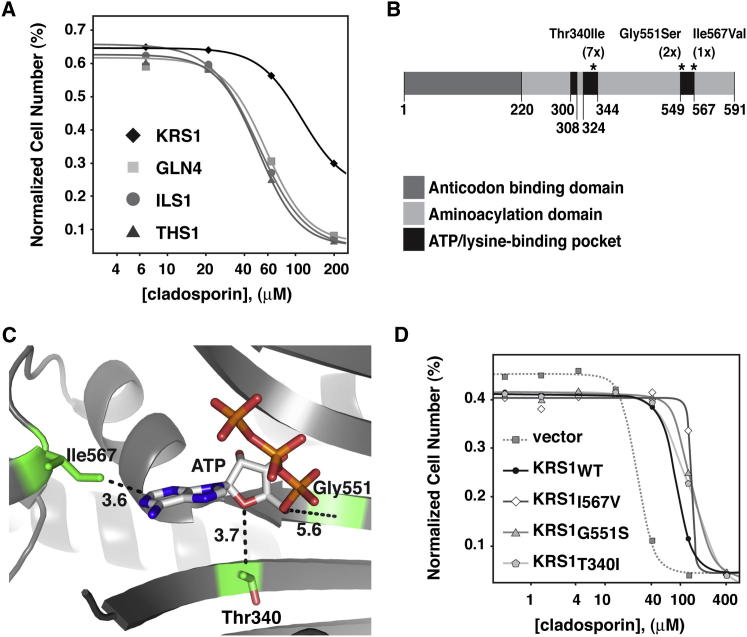
Krs1 Target Validation by Overexpression Analysis and Chemical Mutagenesis of *S. cerevisiae* (A) Cladosporin specificity was evaluated in strains overexpressing the following aminoacyl-tRNA synthetase genes: lysine (*KRS1*), glutamine (*GLN4*), isoleucine (*ILS1*), and threonine (*TRS1*). *KRS1* overexpression confers a 3-fold increase in cladosporin resistance, whereas no change in cladosporin potency was observed in strains overexpressing the other synthetases. (B) Schematic view of the *S. cerevisiae* lysyl-tRNA synthetase (*Sc*Krs1) protein domain organization. Resistance-conferring mutations (asterisks) are labeled and frequency of mutation provided in parentheses. (C) The ATP/lysine binding pocket of the *Sc*Krs1 homology model (gray) is shown. See also Figure S2. The distance (given in angstroms [Å]) between the three mutated residues (stick representations) and atoms in the ATP molecule is indicated next to the dashed lines. (D) Expression of the *Sc*Krs1 mutants Thr340Ile (Krs1_T340I_), Gly551Ser (Krs1_G551S_), Ile567Val (Krs1_I567V_) shifted cladosporin IC_50_ values ∼5.3-fold compared to an empty vector control strain, whereas overexpression of wild-type *Sc*Krs1 (Krs1_WT_) showed a 3.3-fold shift (comparable shift to that in A).

To further test if cladosporin specifically inhibits lysyl-tRNA synthetase, *KRS1* was placed downstream of a Gal1 promoter and transformed into wild-type yeast. The same was done for genes encoding isoleucyl-tRNA synthetase (*IlS1*), glutamyl-tRNA synthetase (*GLN4*), and threonyl-tRNA synthetase (*THS1*). These strains were subsequently tested for growth defects in the presence of serially diluted cladosporin concentrations using galactose as a sole carbon source to drive promoter activity. Only the transgenic strain harboring the plasmid with the *KRS1* gene conferred increased cladosporin resistance (2.2-fold shift), whereas the transgenic strains expressing other aminoacyl-tRNA synthetases (AARSs) exhibited no change in their sensitivity to cladosporin (Figure 2A). In the absence of cladosporin, all strains grew at an equal rate and concurrently reached the same final OD_600_, indicating the overexpression constructs did not have a discernible effect on growth.

### Cladosporin Resistance in Yeast Is Mediated by Mutations in the Active Site of Krs1

To further strengthen the observed genetic link between the *S. cerevisiae* lysyl-tRNA synthetase (*Sc*Krs1) and cladosporin, we examined mutations that confer cladosporin resistance in yeast. Yeast cells were mutagenized with ethylmethanesulfonate and then selected against 50 μM cladosporin, a concentration known to completely inhibit growth of wild-type strains in synthetic complete media (Table S2). Direct sequencing of the *KRS1* gene in 37 clones revealed nonsynonymous mutations in ten of these clones (Figure 2B and Figure S2A).

To better understand the significance of these mutations, we undertook homology modeling. The crystal structures of lysyl-tRNA synthetase from human (Guo et al., 2008) and two bacteria (Sakurama et al., 2009) have been published, and comparison reveals a high degree of structural conservation (Figure S2B). We took advantage of this conservation and used the human crystal structure as a template to generate a *Sc*Krs1 homology model. The *Sc*Krs1 model predicted that the identified mutations, Ile567Val (one strain), Gly551Ser (two strains), and Thr340Ile (seven strains), map to or near the binding pocket for ATP in *Sc*Krs1 (Figures 2B and 2C and Figure S2A). Backcrossing of the ten selected mutants to isogenic wild-type strains of the opposite mating type and subsequent resistance assays revealed that all mutations were dominant. Subcloning of each of the mutated *KRS1* genes into a low copy plasmid and reintroduction into the wild-type strain was sufficient to confer increased resistance to cladosporin (∼5-fold increase), indicating that second-site mutations did not contribute to resistance. We conclude that the mutated *KRS1* gene is responsible for resistance in *S. cerevisiae* and that the resistance phenotype is dominant to the wild-type allele (Figure 2D).

### Amplification of the Lysyl-tRNA Synthetase Locus Confers Resistance in Parasites

**Figure 3:**
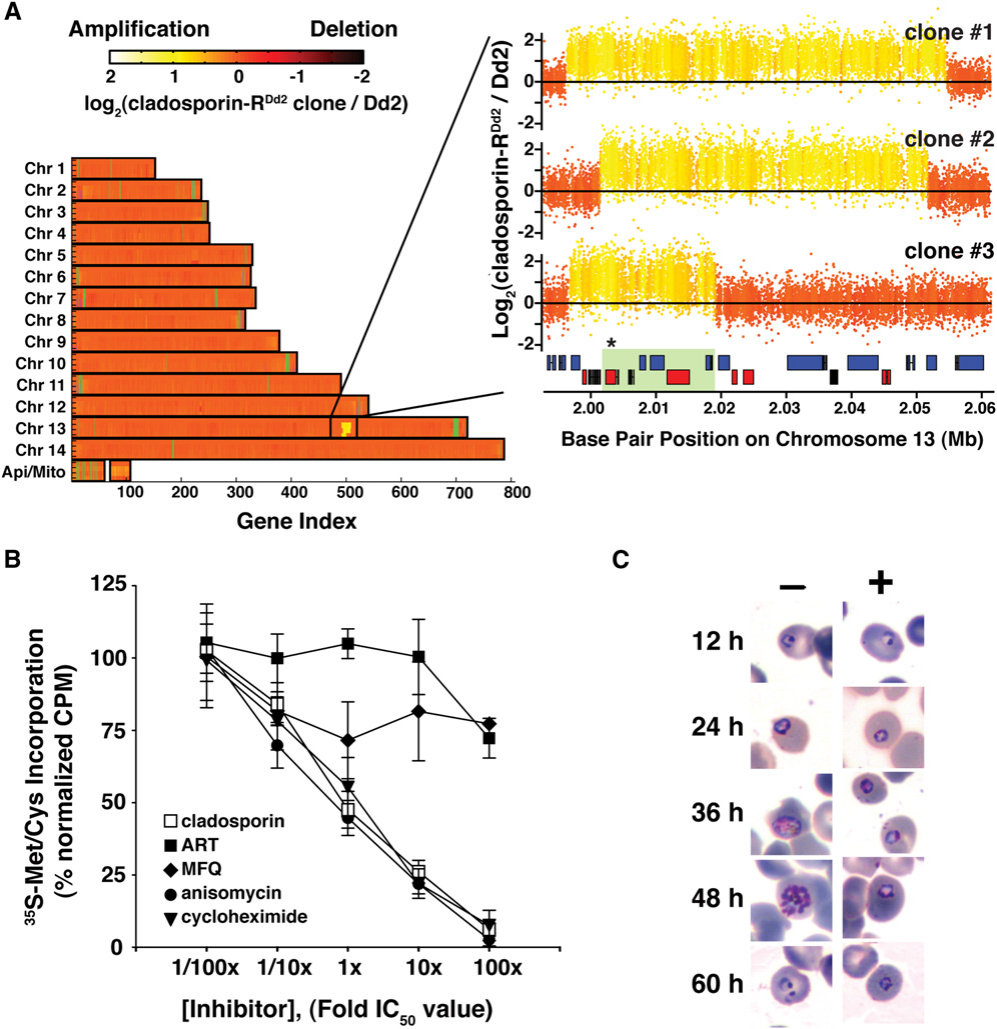
*P. falciparum* Acquires Copy Number Variants in Lysyl-tRNA Synthetase and Has Protein Synthesis Defects in the Presence of Cladosporin (A) Whole-genome analysis of cladosporin-resistant *P. falciparum* clones on a high-density DNA tiling microarray revealed that a common gene locus on chromosome 13 was amplified in each clone. Six genes were shared by all amplification events: lysyl-tRNA synthetase (PF13_0262), small nuclear ribonucleoprotein (MAL13P1.253), a conserved Plasmodium protein of unknown function (PF13_0263), ubiquitin-activating enzyme E1 (PF13_0264), a conserved Plasmodium protein of unknown function (MAL13P1.254), and N6-adenine-specific methylase (MAL13P1.255). An enhanced probe-by-probe analysis of this locus is shown for each clone, and common genes are shaded green. The lysyl-tRNA synthetase gene (asterisk) is present in all amplification events. See also Table S3. (B) Mixed erythrocytic-stage parasites were treated for 1 hr with cladosporin, artemisinin (ART), mefloquine (MFQ), anisomycin, or cycloheximide over a five-log range of drug concentrations to determine their effect on ^35^[S]-cysteine/methionine incorporation. Radioactive counts were normalized to untreated cells, and each data point was plotted as the mean of two experiments performed in triplicate. Error bars represent the standard deviation. (C) The specific time of action for cladosporin in erythrocytic-stage parasites was determined by treating double-synchronized parasites (6 hr interval) and monitoring the cultures over a 60 hr period. The morphology of untreated parasites (–) and parasites treated with 400 nM cladosporin (+) were monitored by Giemsa-stained thin blood smears for one complete 48 hr life cycle (12 hr, ring; 24 hr, trophozoite; 36 hr, early schizont; 48 hr, mature schizont) and the first 12 hr of the second generation. Representative images are shown for each time point. Controls are shown in Figure S3.

To establish target conservation in *P. falciparum*, we evolved cladosporin-resistant parasites (Table 1 and Tables S3 and S4) with hopes that the parasites would acquire resistance mutations in the target gene. The genomes of the cladosporin-sensitive parental clone and cladosporin-resistant clones were compared using a microarray that has the capacity to reveal most genomic changes that are acquired by the resistant strains, including copy number variants, deletions, small insertion/deletion events, and single-nucleotide polymorphisms (Dharia et al., 2009). Since background mutations may appear at a low frequency, the selection was run in triplicate in order to distinguish resistance-conferring mutations from random mutations. Genome scanning revealed that each of the independent clones had acquired copy number variants that shared six genes in a syntenic region of chromosome 13: MAL13P1.253, PF13_0263, PF13_0264, MAL13P1.254, and MAL13P1.255 and PF13_0262, encoding the cytoplasmic lysyl-tRNA synthetase (*Pf*Krs1), the ortholog of *KRS1* (Figure 3A and Table S3). Careful examination of the hybridization data showed that none of the other ∼5,500 genes were mutated in all three clones and that the only genomic differences between the sensitive and mutant lines were the copy number variants that encompassed *Pf*Krs1.

### Cladosporin Blocks De Novo Protein Biosynthesis in *P. falciparum*

Although the high amino acid sequence conservation between the fungal and plasmodial enzymes (Figure S2A) suggests that cladosporin is likely targeting cytoplasmic lysyl-tRNA synthetase in both species, it is possible that one of the other genes in the amplification event could encode the target. Because inhibition of cytoplasmic lysyl-tRNA synthetase is expected to impair protein biosynthesis, incorporation of radioactive amino acids was investigated in cladosporin-treated parasites (Figure 3B). Treatment with cladosporin, or known protein synthesis inhibitors, anisomycin and cycloheximide, caused a rapid, and comparable, drop-off in incorporation of radiolabeled amino acids and indicated protein synthesis inhibition. In contrast, mefloquine and artemisinin, antimalarial drugs with unrelated mechanisms of action, did not diminish radioisotope incorporation, as previously shown (Rottmann et al., 2010).

### Cladosporin Inhibits Cytoplasmic Protein Biosynthesis in *P. falciparum*

Malaria parasites have two lysyl-tRNA synthetases, one of which is targeted to the apicoplast (PF14_0166), a specialized subcellular organelle involved in fatty acid and isoprenoid biosynthesis, while the other one is targeted to the cytoplasm (PF13_0262). The apicoplast is essential to parasite viability, and inhibition of protein synthesis in this organelle yields a characteristic “delayed death” phenotype in which parasite death is observed one generation after drug treatment. Known apicoplast inhibitors include mupirocin, which targets the apicoplast-targeted isoleucyl-tRNA synthetase (Istvan et al., 2011); tetracycline (Goodman et al., 2007); azithromycin (Sidhu et al., 2007); and the apicoplast 23S ribosomal RNA inhibitor clindamycin (Dharia et al., 2009). To distinguish between these two potential targets, *P. falciparum* parasites were synchronized to the ring stage, treated with cladosporin, and the maturation of the parasite monitored over two life cycles. Parasites treated with cladosporin did not exhibit a delayed death phenotype and quickly arrested in the metabolically active trophozoite stage (Figure 3C). The phenotype of cladosporin-treated parasites is indistinguishable from that of anisomycin, another eukaryotic protein synthesis inhibitor, and supports the notion that both have the same mechanism of action. In contrast, clindamycin, which specifically inhibits protein biosynthesis in the apicoplast, did not diminish growth until the end of the second blood-stage life cycle (Figure S3). Although these results do not preclude cladosporin as also targeting the apicoplast lysyl-tRNA synthetase, these data show that cladosporin is a fast-acting agent that shows the same characteristics as other known cytoplasmic protein synthesis inhibitors.

### Cladosporin Is a Potent and Selective Inhibitor of *P. falciparum* Lysyl-tRNA Synthetase

**Figure 4:**
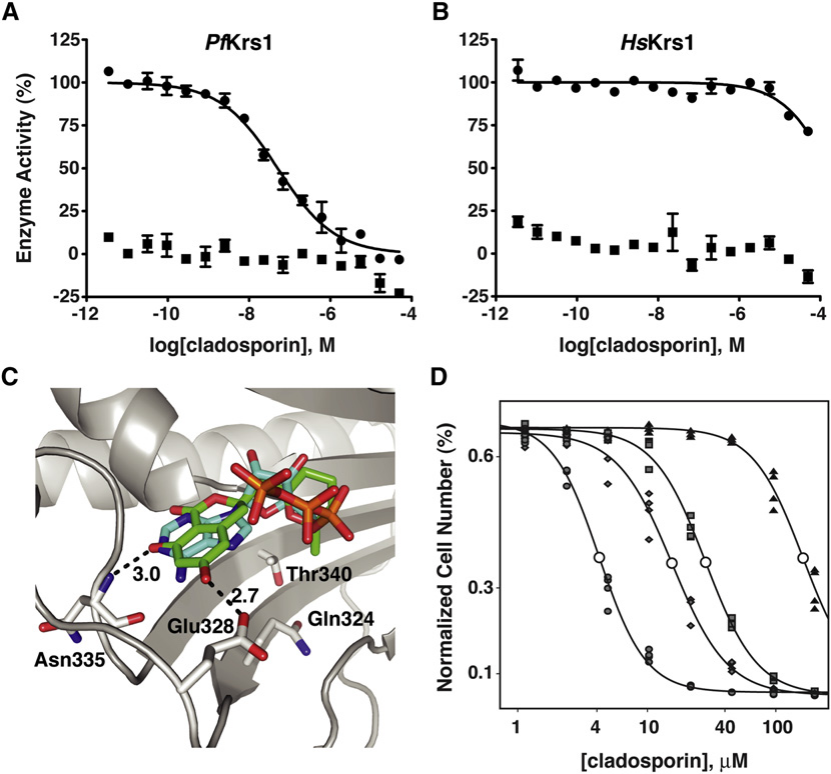
Cladosporin Is Highly Selective for *Pf*Krs1, which Is Modulated in Part by Two Key Active Site Residues (A and B) Direct biochemical analysis of recombinant lysyl-tRNA synthetase reveals that cladosporin has low nanomolar inhibition (IC_50_ = 61 nM) against *Pf*Krs1 (A, squares) and high micromolar activity (IC_50_ > 20 μM) against human Krs1 (B, squares). As a control, recombinant enzyme was assayed under the same conditions but in the absence of the tRNA^Lys^ substrate (circles). Enzymatic data were from at least two independent assays performed in triplicate and expressed as means ± SD. A nonlinear regression curve fit is shown for each. (C) Superimposed structures of cladosporin (green) and ATP (cyan) docked to the yeast homology model (gray). Oxygen and nitrogen atoms are colored red and blue, respectively, in all molecules. Cladosporin is predicted to bind in the ATP-binding pocket of the yeast homology model with the isocoumarin moiety located in the same region as the adenine of ATP. Also, cladosporin's pyrane moiety superimposes between the sugar and the phosphate of ATP and projects toward Gln324 and Thr340. The two hydroxy groups of cladosporin are predicted to form hydrogen bonds with Asn335 and Glu328 (dotted lines; bond length label given in Å). (D) *Sc*Krs1-Gln324Val mutant (IC_50_ = 28 μM; squares) and *Sc*Krs1-Thr340Ser mutant (IC_50_ = 16 μM; diamonds) mimic the differences in the *Plasmodium* ATP pocket and significantly increase the potency of cladosporin compared to wild-type *S. cerevisiae* strain (IC_50_ = 163 μM; triangles). The double mutant, *Sc*Krs1-Gln324Val/Thr340Ser (IC_50_ = 4 μM; circles), which more closely resembles *Pf*Krs1, is 41-fold more sensitive to cladosporin. See also Figure S4. Enzymatic data were from at least two independent assays performed in triplicate and expressed as means ± SD. A nonlinear regression curve fit is shown for each.

To confirm that cladosporin is an inhibitor of *Pf*Krs1, direct biochemical assays were performed on recombinant protein. *Pf*Krs1 activity was assayed in the presence or absence of the corresponding lysine-tRNA (tRNA^Lys^) substrate, and synthetase activity was measured using a Transcreener adenosine monophosphate (AMP) assay. This assay is a far-red, competitive fluorescence polarization immunoassay that detects the reaction product, AMP. Cladosporin possessed a half inhibitory concentration of 61 nM against *Pf*Krs1 (Figure 4A), which is comparable to its activity in cellular screens (IC_50_ 40–90 nM; Table 1). As expected, no activity was observed in the absence of exogenous tRNA^Lys^. The agreement between the biochemical and biological inhibition constants further supports the notion that lysyl-tRNA synthetase is the primary target within the cell.

Our data show that cladosporin has relatively little activity on human cells, including the HepG2-CD81 cells used in the hepatic-stage screen (Table 1). To confirm that cladosporin is selective for *Pf*Krs1, the human counterpart was assayed. Recombinant human lysyl-tRNA synthetase was assayed as described above. As predicted, cladosporin activity was only weakly detected at the high micromolar ranges for human Krs1 (Figure 4B). These data reinforce the notion that cladosporin specifically and selectively targets *P. falciparum* lysyl-tRNA synthetase.

### Structural Basis for Selectivity and Specificity in *Plasmodium* Krs1

The *S. cerevisiae* and *P. falciparum* amino acid sequences share a high degree of identity to the human protein (yeast 58.3%, plasmodium 53.4%), and the homology model could accommodate both primary sequences into the human template with high confidence. According to the predicted models, 30 of 36 amino acids within a 5 Å radius of the ATP/lysine-binding site are conserved between *S. cerevisiae* and *P. falciparum*. Using an in silico docking approach it was possible to model cladosporin into the ATP-binding pocket in an energetically favorable mode, mimicking that of ATP (Figure 4C). Residues Asn335 and Glu328, which hydrogen bond to ATP, are also predicted to hydrogen bond with the hydroxyl groups of cladosporin. The accuracy of this prediction was validated biochemically. Characterization of the recombinant *Pf*Krs1 revealed that lysine had no effect on cladosporin inhibition (Figure S4A), whereas increasing concentrations of ATP in the reaction buffer significantly reduced cladosporin inhibition (Figure S4B). This was corroborated by the inability of exogenous lysine in culturing media to affect cladosporin potency against yeast cells (Table S5). Taken together, these data support the notion that cladosporin interacts with the ATP-binding pocket of lysyl-tRNA synthetase and shares a similar mode of binding to ATP.

To further understand the basis of cladosporin selectivity, the amino acid residues of the ATP-binding pocket were compared to those in Krs1 of other species (Figure S4C). While the majority of amino acid residues in the binding pocket were highly conserved, a clear divergence existed at two amino acid positions corresponding to *S. cerevisiae* residues Gln324 and Thr340 (Table 2). In *Plasmodium* spp., these positions are occupied by a valine and serine residue, respectively. Based on the identity of the *Plasmodium* amino acid residues, smaller side chains are favored, presumably due to decreased steric hindrance. Additionally, a hydrophobic residue at position 324 also appears favorable for cladosporin binding.

Despite being separated by 15 amino acids in the primary sequence, the homology model predicted that these residues were juxtaposed in the active site (Figure S4D). The docking model also predicts that the pyrane moiety of cladosporin points toward both of these residues and that Thr340 resides at an intimate distance of 3.7 Å (Figure 4C and Figure S4D). Extending the comparison to other eukaryotic and prokaryotic pathogens with Krs1 orthologs, a clear correlation exists between cladosporin activity and the identity of the amino acids at these two key positions in the ATP-binding pocket (Table 2). The trend predicts a significant loss in cladosporin potency whenever a bulkier residue (replacement of serine with threonine) is present at position 340. If this mutation is accompanied by a modestly more bulky and polar residue at position 324 (valine to glutamine or asparagine), then an even more significant decrease in activity is observed. The presence of a methionine at position 340 in bacteria species, which would likely impose an even greater steric hindrance than threonine, leads to the most significant loss in cladosporin potency.

**Figure 5:**
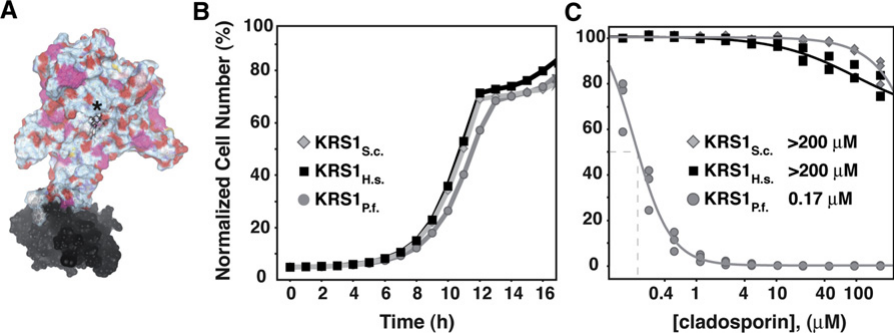
Yeast Cells Dependent on Chimeric Lysyl-tRNA Synthetases Exhibit Wild-Type Growth Kinetics but Show Differential Sensitivity to Cladosporin (A) The homology model of *Sc*Krs1 with cladosporin docked into the ATP binding site (asterisk). Amino acids 1–220 corresponding to the tRNA-binding domain are colored black, whereas residues beyond 220, which correspond to the aminoacylation domain, are shown in color. (B) Growth curves of yeast cells solely dependent on heterologous, chimeric lysyl-tRNA synthetases with aminoacylation domains of the indicated species. (C) Cladosporin dose-response curves of yeast cells solely dependent on heterologous, chimeric lysyl-tRNA synthetases with aminoacylation domains of the indicated species. Data for (B) and (C) were collected using the optical density assay and are a representation of triplicate experiments. The independent data points for each experiment are shown. In (C), a nonlinear regression curve fit was performed on the means ± SD of these data.

To validate whether *Sc*Krs1 positions 324 and 340 are significant factors in cladosporin selectivity and specificity, these positions were mutated in yeast to mimic those residues found in *Plasmodium* spp. Substantial increases in cladosporin potency were observed for both mutations. The single mutation Gln324Val produced a 5.7-fold increase in cladosporin sensitivity, whereas an even more pronounced increase by 10.4-fold was observed for the Thr340Ser mutant (Figure 4D). As expected, the double mutant Gln324Val/Thr340Ser led to an even greater sensitization of yeast to cladosporin (38.7-fold increase), as the active site of this mutant more closely resembled that in *Plasmodium* spp. (Figure 4D). Finally, because mutating two residues might alter the efficiency of the active site, we replaced the entire yeast aminoacylation domain with those from either human or *P. falciparum* (Figure 5A). These strains were engineered to be dependent on the hybrid synthetases, and all showed normal growth (Figure 5B). However, the *S. cerevisiae* strain bearing the *P. falciparum* hybrid synthetase showed a 1,000-fold increase in sensitivity to cladosporin (IC_50_ from >200 μM to 0.17 μM, Figure 5C). In contrast, the strains bearing the human or yeast synthetases showed little change in cladosporin sensitivity (Figure 5C). These data demonstrate that the conformation of the aminoacylation domain renders the malaria parasite protein uniquely susceptible to cladosporin and that lysyl-tRNA synthetase is the primary target of cladosporin.

## Discussion

Cell-based screening has recently been shown to be an attractive way to find new leads for malaria drug development (Gamo et al., 2010; Guiguemde et al., 2010; Plouffe et al., 2008). In this respect, cladosporin could be an attractive lead, given the species selectivity and potent nanomolar activity against liver-stage parasites. Unfortunately, cladosporin possesses poor oral bioavailability, a key requirement for an antimalarial. Therein lies a key disadvantage of the cell-based lead-finding approach—if an initial hit is chemically intractable and the target is not known, there may be no path forward. However, if a chemically validated target can be discovered, then a more focused and extensive lead-finding biochemical screen can be readily initiated.

Methodological choices for finding targets in malaria parasites have been traditionally limited. Cheminformatic predictions based on historical screening data against different biochemical targets (Gamo et al., 2010) and/or in silico docking typically only creates hypotheses, and extensive, time-consuming experimentation is required for confirmation. Affinity chromatography, a method by which an inhibitor is immobilized to resin and incubated with cell lysate to recover the molecular target, has been used with limited success (Kato et al., 2008; Knockaert et al., 2000). However, nonspecific binding, variable parasite protein target abundance (1,000-fold differences in protein abundance have been observed), and limitations in generating enough parasite lysate for analysis make this a challenging approach. Thus, evolution of drug-resistant parasites combined with genome scanning (Dharia et al., 2009) is our preferred method for discovering the target of antimalarials (McNamara and Winzeler, 2011; Meister et al., 2011; Nam et al., 2011; Rottmann et al., 2010). Nevertheless, some ambiguity often remains. Specifically, more than one gene may be mutated, or in some cases, resistance is conferred by a copy number variant encompassing several genes. The use of yeast deletion strains to complement the parasite evolution studies provides a powerful method of confirmation. However, an inherent disadvantage of genetic methods in both yeast and parasites is that only a resistance mechanism may be revealed, and thus additional experiments will always be necessary to validate the mechanism of action.

While it is possible that the amplification in lysyl-tRNA synthetase only confers resistance to cladosporin, or that cladosporin affects its killing through a different mechanism than by inhibiting lysyl-tRNA synthetase in *P. falciparum*, our biochemical evidence suggests that lysyl-tRNA synthetase is the primary target of cladosporin. Recent evidence suggests that human Krs1 has diverse biological roles beyond protein synthesis. Among these functions, it is secreted as a proinflammatory cytokine (Park et al., 2005) and is an integral component of a multimeric protein complex that affects regulatory signal pathways (reviewed in Park et al., 2010). The large therapeutic window between species makes inhibition of human Krs1 a minor concern. However, it is not known whether these same diverse functions are conserved in *Plasmodium* spp. Therefore, it is presently unclear if specifically targeting *Pf*Krs1 and/or potential components in these putative pathways makes this enzyme a more desirable parasite target than other AARSs.

Aside from the noncanonical functions, selective inhibition of the protein translational function of *Pf*Krs1 has obvious value because of the significant parasite proliferation during both liver- and blood-stage infections. Indeed, AARSs have recently gained recognition as desirable targets in antibacterial drug discovery programs (Hurdle et al., 2005). For instance, mupirocin has been shown to be a selective inhibitor of bacterial isoleucyl-tRNA synthetase (Hughes and Mellows, 1980; Sheng and Zhang, 2011), and a series of diaryl diamines specifically target *Trypanosoma brucei* methionyl-tRNA synthetase (Shibata et al., 2011). Also, a broad-spectrum inhibitor of fungal leucyl-tRNA synthetases, AN2690, is in development for the treatment of onychomycosis (Rock et al., 2007). However, targeting the AARS of an invasive micro-organism without inhibiting the human counterpart is a major challenge in drug discovery. Unlike the highly divergent prokaryotes, eukaryotic pathogens such as fungus (*Candida* spp.) and apicomplexan parasites (*Plasmodium*, *Toxoplasma*, and *Trypanosoma* spp.) generally have higher conservation with the homologous human AARS. Moreover, the lack of three-dimensional structures from some eukaryotic pathogens limits rational structure-based drug design to help identify and exploit differences. Despite this, we show here that selective eukaryotic AARS inhibitors can be achieved, opening the door for target-based drug discovery efforts focused on lysyl-tRNA synthetase. This in turn may lead to small molecules with the pharmacokinetic and pharmacodynamic properties needed for the orally bioavailable antimalarial drugs needed to support global eradication efforts.

## Experimental Procedures

### HIP Profiling

Cladosporin was tested in three independent HIP experiments at 110 μM (two times) and 150 μM (one time). Each experiment identified the *krs1/KRS1* strain as the most sensitive strain. The experimental execution and processing of results were based on previous protocols (Pierce et al., 2007). The HIP assay was performed in 24-well plates with 1,600 μl of YPD per well. Per experiment, cladosporin was tested in two wells and compared to eight wells with DMSO. At the beginning of each experiment, 100 μl per well of a 1.5 OD_600_/ml log phase preculture comprising all strains of the heterozygous deletion collection strains were added (OpenBiosystems, part numbers YSC1056). Plates were incubated for 16 hr in a robotic shaking incubator at 30°C/55 RPM, allowing for approximately five doublings and then a robotic system back-diluted 50 μl/well into fresh medium with compound. This procedure was repeated two more times until the yeast had doubled 20 times. The HIP pool construction, gDNA extraction, hybridization procedure, and calculation of sensitivity scores were based on the methods published by Pierce et al. (Pierce et al., 2007). In addition to the sensitivity score, for each gene, a z score was calculated from the TAG4 microarray intensities by taking the logarithm of the ratio of mean experiment versus mean control tag-averaged intensities. The z scores of all genes were then robustly normalized experiment- and gene-wise.

### *S. cerevisiae* Aminoacyl-tRNA Synthetase Overexpression

Lysyl-tRNA synthetase (*KRS1*), isoleucyl-tRNA synthetase (*IlS1*), glutamyl-tRNA synthetase (*GLN4*), and threonyl-tRNA synthetase (*THS1*) were obtained from the previously characterized yeast GAL overexpression collection (Gelperin et al., 2005) (OpenBiosystems, part numbers YSC3870). Cells were grown in synthetic medium supplemented with 2% galactose and serial dilutions of cladosporin (200 μM max concentration, 3:1 dilution). DMSO was normalized to 2%. Dose-response curves were calculated by taking the 20 hr OD_600_ measurements and applying a logistic regression curve fit.

### Selection of Drug-Resistant *S. cerevisiae*

Strain BY4743Δ8, derived from BY4741 but deleted for eight genes involved in drug resistance (efflux pumps, *SNQ2*, *PDR5*, *YOR1*; transcription factors, *PDR1*, *PDR2*, *PDR3*, *YAP1*, *YRM1*) was incubated in 2.5% ethylmethanesulfonate until only 50% of the cells formed colonies. A total of 2 × 10^7^ mutagenized CMB970 cells were plated on two 14 cm dishes with synthetic complete medium (0.7 g/l Difco Yeast Nitrogen Base without amino acids, 0.79 g/l MPbio CSM amino acid mixture, 2% glucose) containing 50 μM cladosporin. After 4 days, 37 resistant colonies appeared. Resistance was confirmed by restreaking on 50 μM cladosporin. Genomic DNA was extracted (YeaStar Genomic DNA Extraction Kit, Zymo Research), and the *KRS1* gene including promoter and terminator was sequenced (P1, CCTTACCTGAAGAGAAGG; P2, GGTCAAGGAGATCACTGG; P3, CATCCCTTATCCCTTCTC; P4, CTCGACACCAACGATATC). Of these 37, ten colonies contained nonsynonymous mutations in the *KRS1* open reading frame. To distinguish between recessive and dominant mutations, the ten strains were crossed with an isogenic wild-type strain of opposite mating type and resistance confirmed by restreaking on 50 μM cladosporin. All ten strains resulted in resistant diploids. To exclude secondary mutations, *KRS1* genes bearing three different mutations were cloned into the SmaI and KpnI sites of the single copy pRS416 plasmid by PCR amplification with primers, containing restriction sites at their 5′ ends (KRS1-SmaI, ATAATACCCGGGACCCATGAGCACAATAG; and KRS1-KpnI, ATAATAGGTACCAGCGTATAGCACATCCAC) and transformed into cladosporin-sensitive BY4741 yeast. Resistance was confirmed by recording dose-response curves in YPD medium with serial dilutions of cladosporin (200 μM max concentration, 3:1 dilution). DMSO was normalized to 2%. Curves were calculated by taking the 16 hr OD_600_ measurements and applying a logistic regression curve fit.

### Generation and Assay of Mutant *KRS* Lines in Yeast

*S. cerevisiae KRS1* wild-type gene was cloned into a pRS416 low copy number plasmid. Single mutations were introduced by site-directed mutagenesis, and the double mutant was generated by excising the 623 bp fragment containing the Gln324Val mutation and cloning it between the EcoRI sites of the Thr340Ser-containing plasmid. The resulting plasmids were verified by direct DNA sequencing, transformed into *KRS1*/*krs1* heterozygous deletion mutants, sporulated, and the tetrads dissected. Selecting for uracil auxotrophy and G418 resistance identified colonies exclusively dependent on the plasmid encoding mutant *KRS1*. IC_50_ values were determined by testing growth of four independent colonies in SD-ura medium with 12-point serial dilutions at a dilution factor of 3. The top concentration of cladosporin was 200 uM and resulted in a final DMSO concentration of 2%. Growth was monitored by reading OD_600_ values over 24 hr, and these data were fit to a nonlinear regression by Prism to calculate the IC_50_ value.

### Selection of Drug-Resistant Malaria Parasites

Cladosporin-resistant parasites were selected using the protocol described in Rottmann et al. (Rottmann et al., 2010). In brief, clonal Dd2 parasites were established in triplicate flasks and evolved independently. Drug challenge was initiated at the IC_50_ value for cladosporin (40 nM) and increased in 10–40 nM steps. The continuous exposure and challenge to sublethal concentrations of inhibitor were carried out for 2 months, with final drug concentration between 380 and 400 nM. Clonal cladosporin-resistant clones were selected by limiting dilution using complete media supplemented with 340 nM cladosporin. Each clone was tested against a panel of antimalarial compounds (Table S4).

### Genome Scanning of *P. falciparum* gDNA

Genomic DNA samples, extracted from cladosporin-sensitive and cladosporin-resistant clones, were digested, labeled, and analyzed as previously described by Dharia et al. (Dharia et al., 2009). Briefly, 15 μg of digested biotin end-labeled genomic DNA was hybridized to a custom high-density tiling array (Pftiling). PfGenominator version 2.0 (freely available at http://www.scripps.edu/winzeler/software/) was used to perform a comparative genomic analysis to detect hybridization differences between the parental Dd2 clone and each of the independently derived cladosporin-resistant clones. Modular analyses were performed to identify copy number variation and single nucleotide polymorphism (SNP) events. A p value of cutoff <1 × 10^−8^ was enforced on the SNP analysis to filter out false positives, and a probe-by-probe analysis was performed on each SNP to validate its likelihood.

### Metabolic Labeling with [^35^S]-Methionine/Cysteine for Protein Synthesis Inhibition Studies

A metabolic labeling assay was performed according to the protocol described in Rottmann et al. (Rottmann et al., 2010). For more information, refer to the Supplemental Experimental Procedures.

### Molecular Modeling

Structural alignment of the X-ray crystal structures of human (Protein Data Bank [PDB] ID 3BJU) and bacterial lysyl-tRNA synthetases (PDB ID 1BBU and 3E9H) using ICM software (Molsoft, LLC) revealed a high degree of similarity (see Figure S4). The yeast homology model was built using the MOE software (Chemical Computing Group). The human lysyl-tRNA synthetase X-ray structure showed the greatest amino acid conservation to the yeast sequence and was therefore selected as the template for the yeast homology model. The homology model was built using MOE default parameters. Prior to docking studies with cladosporin, a model for the binding of ATP in the yeast protein was created. ATP was manually placed within the yeast homology model, and the protein side chains were minimized in order to best accommodate this compound. Cladosporin was docked in this protein using Glide SP (Schrödinger, LLC) and refined using the MM/GBSA method implemented in Prime (Schrödinger, LLC).

### Cloning and Expression of *Plasmodium* Lysyl-tRNA Synthetase

The sequence of the lysyl-tRNA synthetase (PF13_0262) was optimized for *E. coli* codon usage and synthesized in pUCminus vector (1st Base). The gene was subcloned into pET-28a(+) expression vector (Novagen) using BamHI and EcoRI sites, and transformed into *E. coli Tuner* (DE3) cells (Novagen). Of 2xYT media, 2 L containing 50 μg/ml kanamycin was inoculated with 20 ml of overnight culture and grown at 37°C. Expression was induced with 0.1 mM IPTG at A_600_ = 0.8 and incubated for 24 hr at 13°C. The cells were harvested by centrifugation at 6,000 rpm for 10 min and frozen at −80°C.

### Purification of *Plasmodium* Lysyl-tRNA Synthetase

Frozen cell pellets were thawed and resuspended in 60 ml cold lysis buffer (20 mM Tris HCl [pH 8.0], 500 mM NaCl, 0.5 mM TCEP) supplemented with 0.01% SDS and a protease inhibitor tablet (Roche Diagnostics). Cells were lysed by sonication, and the crude lysate was centrifuged (22,000 g, 1 hr at 4°C). The supernatant was clarified through a 0.22 μm filter (Millipore) and loaded onto a Ni-affinity column. The column was washed with 10 column volumes wash buffer (20 mM Tris [pH 8.0], 500 mM NaCl, 50 mM imidazole, and 0.5 mM TCEP) and the protein eluted with a gradient profile of 50–500 mM imidazole over 20 column volumes. The protein-containing fractions were dialyzed against low-salt buffer (20 mM Tris [pH 8.0], 100 mM NaCl, and 0.5 mM TCEP) and loaded to a MonoQ 5/50GL column (GE Healthcare). The column was washed with 4 column volumes of wash buffer 2 (20 mM Tris [pH 8.0], 150 mM NaCl, 0.5 mM TCEP). A shallow gradient elution was carried out with 150–1,000 mM NaCl over 50 column volumes. A final purification step was repeated with the Ni-affinity column. The concentration of the eluent-containing target protein was measured by absorbance at 280 nm with ε = 41,780 M^−1^ cm^−1^.

### Lysyl-tRNA Synthetase Activity Assay

Purified recombinant human enzyme was purchased from Origene (catalog number TP300311). Enzymatic aminoacylation by lysyl-tRNA synthetase was assayed with the Transcreener AMP assay system (BellBrook) in a Corning 384-well black plate. The *Pf*tRNA^Lys^ substrate was chemically synthesized (Trilink). The assay was performed in a 10 μl volume containing either 250 nM of *Plasmodium* purified enzyme or 30 nM of human lysyl-tRNA synthetase (OriGene) in 40 mM HEPES (pH 7.5), 1 mM DTT, 40 μM ATP, 10 μM *Pf*tRNA^Lys^, 8 mM MgCl_2_, and 40 μM L-lysine. Reagent mixing was performed on orbital shaker for one minute at 900 rpm, followed by incubation at RT for 4 hr. Enzymatic activity was terminated by the addition of an equal volume (10 μl) of detection reagent and yielded a final concentration of 0.5× stop buffer, 1 nM AMP-Alexa633 tracer, and 2.5 μg/ml ADP antibody. Fluorescence polarization measurements were performed on the Infinite M1000 plate reader (Tecan) using a 635 nm excitation and 680 nm emission (20 nm bandwidth) filter settings. IC_50_ values were determined by subtracting the background activity and performing nonlinear regression with Prism (GraphPad).

### Construction of Hybrid Synthetase Yeast Strains

Protein sequences of *Homo sapiens* (NM_001130089) and *P. falciparum* lysyl-tRNA synthetase (PF13_0262) were aligned with *S. cerevisiae* Krs1 sequence. The first 220 *Sc*Krs1 amino acids where taken (tRNA binding domain) and then fused to the aminoacylation domains for the two respective sequences. These hybrid protein sequences were back-translated into DNA using yeast codon usage, and synthetic DNA fragments were created with a SmaI site at the 3′ end, 332 bp of *S. cerevisiae KRS1* promoter sequence, the hybrid *KRS1* sequence, 206 bp of *S. cerevisiae KRS1* terminator sequence, and a KpnI site. As a control, the same construct was generated using the full-length *S. cerevisiae KRS1* sequence. The DNA fragments where then cloned into the cloning site of pBYIntURA plasmids using the SmaI and KpnI sites, linearized with BstX1, and transformed into the heterozygous BY4743 *KRS1/krs1::kanMX4* (*ura3Δ*) strain where the constructs integrated into the TRP1 locus by selecting on medium lacking uracil. Transformants were verified by analytical PCR, sporulated, and dissected. Germinating spores were further analyzed for the integration of the plasmid in the TRP1 locus and presence of the KanMX deletion by analytical PCR. Integrity of the integrated lysyl-tRNA synthetase constructs was verified by sequencing. Growth kinetics of the strains were assessed by measuring OD_600_ values in hourly intervals on a robotic system in 96-well plates. Sensitivity against cladosporin was tested by growing the stains in serially diluted compound as described previously.

Additional methods are provided in the Supplemental Information.

## Figures and Tables

**Table 1:** Cladosporin Activity against *Plasmodium* Blood and Liver Stages and Human Cell Lines

Strain or Cell Line	*Plasmodium* Activity	Cell Line
IC_50_ (nM)	CC_50_ (nM)
3D7^a^	45.4 ± 6.0	
Camp R^a^	77.9 ± 3.2	
D10^a^	89.6 ± 11.4	
D6^a^	72.1 ± 3.1	
K1^a^	80.1 ± 10.3	
NF54^a^	87.9 ± 5.3	
FCB^a^	66.7 ± 4.9	
FCR3^a^	57.4 ± 10.8	
HEp2^b^		9,666 ± 2,200
HeLa^b^		74,285 ± 18,830
HepG2^b^		43,568 ± 25,080
Huh7^b^		>100,000
HepG2-CD81^b^		>10,000
*P. yoelii* liver schizont^c^	39.1 ± 18.4	
Dd2 clone^1^	62.5 ± 3.5	
Cladosporin-R^Dd2^ clone#1^a^	377.1 ± 31.4	
Cladosporin-R^Dd2^ clone#2^a^	389.5 ± 39.6	
Cladosporin-R^Dd2^ clone#3^a^	374.7 ± 54.0	

a IC_50_ determined by the 72 hr SYBR Green cell proliferation assay.

b Fifty percent cytotoxic inhibitory concentration (CC_50_) was determined by CellTiter-Glo viability assay.

c IC_50_ based on the mean parasite area calculated in the high-content screen.

**Table 2:** Summary of Amino Acid Conservation at Key Residues in the ATP Pocket of Krs1 and Corresponding IC_50_ Value of Cladosporin

Organism	Cladosporin Inhibition		Key Active Site Residues^a^	
IC_50_ (μM)	MIC^b^ (μg/ml)	Position 324	Position 340
*Plasmodium falciparum*^c^	0.04–0.08		Val	Ser
*Plasmodium yoelii*^d^	0.04		Val	Ser
*Trypanosoma brucei*	2.05		Val	Thr
*Leishmania donovani*	2.56		Val	Thr
*Toxoplasma gondii*	2.63		Asn	Ala
*Homo sapiens*	>10		Gln	Thr
*Saccharomyces cerevisiae*	30–110		Gln	Thr
*Escherichia coli*		>100	Asn	Met
*Bacillus stearothermophilus*		>100	Val	Met

a Amino acid identity at homologous positions to *Sc*Krs1 residues 324 and 340 based on primary sequence alignments (see also Figure S4).

b Minimal inhibitory concentrations of cladosporin reported by Anke (1979).

c SYBR Green proliferation assay of blood-stage parasites.

d High-content imaging of liver-stage parasites.

## Supplemental Information

Document S1. Figure S1, Figure S2, Figure S3, Figure S4, Table S1, Table S2, Table S3, Table S4, Table S5, Supplemental Experimental Procedures, and Supplemental References
