# Supplementary material for: Selective and Specific Inhibition of the Plasmodium falciparum Lysyl-tRNA Synthetase by the Fungal Secondary Metabolite Cladosporin
Source: Cell Host Microbe. 2012 Jun 14;11(6):654–63. doi: 10.1016/j.chom.2012.04.015 (PMC3391680; doi:10.1016/j.chom.2012.04.015)
Supplement: Document S1. Figure S1, Figure S2, Figure S3, Figure S4, Table S1, Table S2, Table S3, Table S4, Table S5, Supplemental Experimental Procedures, and Supplemental References [file mmc1.pdf]

Cell Host & Microbe, Volume *11*

Supplemental Information

Selective and Specific Inhibition of the *Plasmodium falciparum* Lysyl-tRNA Synthetase by the Fungal Secondary Metabolite Cladosporin

Dominic Hoepfner, Case W. McNamara, Chek Shik Lim, Christian Studer, Ralph Riedl, Thomas Aust, Susan L. McCormack, David M. Plouffe, Stephan Meister, Sven Schuierer, Uwe Plikat, Nicole Hartmann, Frank Staedtler, Simona Cotesta, Esther K. Schmitt, Frank Petersen, Frantisek Supek, Richard J. Glynne, John A. Tallarico, Jeffery A. Porter, Mark C. Fishman, Christophe Bodenreider, Thierry T. Diagana, N. Rao Movva, and Elizabeth A. Winzeler

Inventory of Supplemental Information

Figure S1. Cladosporin inhibition of *S. cerevisiae* wild type strain BY4743 and a drug efflux mutant strain BY4743Δ8, related to Figure 1.

Description: Provides the reader with plots showing the non-linear regression curve fit for yeast inhibition data. We cite these IC_{50} values in the main text (2nd sub-section: **Chemogenomic profiling reveals cladosporin targets yeast lysyl-tRNA synthetase**) and provides increased transparency to our data quality.

Figure S2. Amino acid and structural similarities amongst Krs1 from different species, related to Figure 2.

Description: Panel A gives additional insights to the amino acid conservation in both organisms at the mutated residues. The resultant alignment is rather large in size to ensure it is legible to readers and thus could not be successfully included in Figure 2. Panel B relates to data presented in the sub-section titled **Structural basis for the parasite enzyme specificity.** Here we state that there is a "high degree of structural conservation" between known Krs1 structures. This figure expands on that statement and provides r.m.s.d. values to the readers as well as visual confirmation of this conservation. This high level of conservation supports our experimental reasoning to create a homology model from which we eventually proceed to use for prediction of key amino acids in selectivity.

Figure S3. Stage of action of cladosporin, anisomycin and clindamycin in synchronized *P. falciparum* parasites, related to Figure 3.

Description: Provides additional images of control compounds that, due to size restrictions, could not be easily included in panel C of Figure 3.

Figure S4. Cladosporin binds the ATP pocket of Krs1 where two amino acids dictate selectivity, related to Figure 4 and Table 2.

Description: Panels A and B provide additional data supporting the notion that cladosporin targets the ATP-binding pocket and not the lysine-binding pocket of Krs1. Panel C provides an amino acid alignment that was critical in assembling Table 2, whereas panel D gives a three-dimensional model of the predicted ATP pocket with bound cladosporin and the corresponding conservation of amino acids within that pocket. Panel D is somewhat redundant to Figure 4C, but offers a slightly different view of cladosporin docked within the van der Waals surface representation of Krs1 ATP-binding pocket. Finally, panel E provides complementary and supporting data that *Sc*Krs1 amino acids 324 and 340 are critical in cladosporin selectivity. Whereas Figure 4D demonstrated that changing these two residues in *Sc*Krs1 to the corresponding *Pf*Krs1 residues increases yeast sensitivity to cladosporin, these data show that changing the *Pf*Krs1 residues to those in *Sc*Krs1 significantly reduce cladosporin potency.

Table S1. Quantification of cidal and static properties of cladosporin against malaria parasites, related to Table 1.

Description: These data don't have a good home within the manuscript, but are most closely associated to Table 1. Table S1 summarizes data on the cidal properties of the compound against *P. falciparum*. We expect these data to be of strong interest to those in drug discovery and support that cladosporin is not a static inhibitor.

Table S2. Potency of cladosporin on yeast strains in variant growth conditions and experiments, related to Figure 1.

Description: Throughout the manuscript we present cladosporin activity in multiple yeast experiments. Cladosporin activity is influenced slightly by factors such as growth media, cladosporin batch and yeast strain. To reduce confusion to the reader and help explain the variations in cladosporin activity, we now provide this comprehensible table detailing which conditions (media, strain, etc) were used in each experiment and the corresponding IC_{50} value of cladosporin.

Table S3. Summary of genome scanning and IC_{50} values in drug-evolved and parental lines, related to Figure 3A.

Description: These data provide additional IC_{50} properties of the cladosporin-resistant parasite lines as well as summarizing the genomic changes observed in each cladosporin-resistant clone.

Table S4. IC_{50} values of cladosporin-R^{Dd2} clones against a panel of antimalarials with unique mechanism of action, related to Figure 3.

Description: This table provides IC_{50} values to seven additional compounds with diverse mechanisms of action and their inclusion in Table 2 would have made for an overwhelming table of values. Instead we only presented the most important of the IC_{50} values in Table 2 and moved the others to SI.

Table S5. Exogenous lysine in growth media does not alter cladosporin activity in yeast, related to Figure 4.

Description: Figure 4 demonstrates that cladosporin inhibition of recombinant *Pf*Krs1 is adversely affected by ATP but not lysine. In support of this observation, we show that high levels of exogenous lysine in the yeast growth media cannot rescue yeast from cladosporin toxicity. Moreover, lysine (even up to 100 mM) has no effect on cladosporin potency. Thus, this is consistent with cladosporin being a competitive inhibitor of ATP in *Sc*Krs1.

SUPPLEMENTAL EXPERIMENTAL PROCEDURES

Statistical analysis of HIP data. Sensitivity was computed as Median absolute deviation logarithmic (MADL) score for each compound/concentration combination as follows: let ID be the intensity of strain Y in a sample that is treated with Compound D and IC the average intensity of the control samples. Furthermore, let mD be the median and madD be the MAD of the logarithmic ratio lnID/IC overall strains for the sample treated with Compound D. The MADL score sY for strain Y is now given by sY = (ln ID/IC – mD) / madD. The aim of the analysis is to detect outliers; these should influence the normalization as little as possible. In addition to the MADL score for each gene, a Z-score was calculated from the TAG4 microarray intensities by taking the logarithm of the ratio of mean experiment versus mean control tag-averaged intensities allowing for 15% outliers. Similarity of the obtained HOP profiles was compared by calculating the Pearson correlation coefficients of a compound HOP profile with all other compound profiles.

Sporozoite hepatocyte invasion assay and immunofluorescence quantification. Inhibition of *P. yoelii* liver stage development was monitored as previously described by Meister *et al.* (Meister et al., 2011). Briefly, HepG2-A16-CD81^{EGFP} cells stably transformed to express a GFP-CD81 fusion protein (Yalaoui et al., 2008), were seeded in 384-well plates (Aurora 384 IQ-EB Black/Clear Plates), 20–26 hours prior to the actual infection. Two hours prior to infection, 50nl of compound in DMSO (0.1% final DMSO concentration per well) were transferred with a PinTool (GNF Systems) into the assay plates (10 µM final concentration). *P. yoelii* sporozoites were freshly dissected from infected *A. stephensi* mosquito salivary glands and filtered twice through a 40 µm nylon pore cell strainer. The HepG2-A16-CD81^{EGFP} cells were then infected

with $8x10^3$ sporozoites per well and the plates spun down. After infection and 2 hours incubation at 37 °C, the cultures were washed, new media and compound added and further incubated with 5-fold increased concentration of penicillin/streptomycin (Sigma-Aldrich) (500 units penicillin and 0.5 mg streptomycin per ml) for 48 hours at 37°C.

After fixing by the addition of 12.5 µl of 20% solution of paraformaldehyde (EMS) and treatment with 0.5% Triton-X-100 (Thermo Fisher Scientific) the exoerythrocytic forms (EEFs) were stained using a mouse polyclonal serum against *Plasmodium* heat shock protein 70 (*Py*HSP70)(Rénia et al., 1990), a DyLight 649 goat anti-mouse IgG, Fcγ fragment specific secondary antibody (Jackson Immuno Research, cat# 115-495-071) and the Hoechst 33342 nucleic acid dye (Invitrogen). Stained EEFs were then quantified using the Opera Confocal High Content Screening System (PerkinElmer). All images were then analyzed using a custom Acapella™ (PerkinElmer) script parametrized for this assay.

Maintenance, synchronization and imaging of erythrocytic stage *P. falciparum* cultures. A clonal stock of the *P. falciparum* line Dd2 (parental clone) was established by limiting dilution from a Dd2 isolate (MRA-156) obtained from the Malaria Research and Reference Reagent Resource Center (MR4; American Type Culture Collection), and used throughout these studies. All *P. falciparum* parasites were cultured in human erythrocytes and complete culturing media (CM) as previously described (Dharia et al., 2009). Highly synchronous cultures were achieved using the sorbitol synchronization method (Lambros and Vanderberg, 1979). Visualization of parasites were performed on Giemsa-stained thin blood smears under a 100x oil immersion objective on a Zeiss Primo Star light microscope. Digital images were captured using a Canon

Powershot G10 camera attached to the microscope and imported in Adobe Photoshop and Illustrator for image sizing and figure generation, respectively.

SYBR Green cell proliferation assay. Compounds were tested for activity against asexual blood stage parasites using a previously described screening method (Plouffe et al., 2008). Briefly, parasites were incubated with 1.25 µM compound for 72 hrs in 384-well plates, and then stained with the nucleic acid binding dye SYBR Green I. Fluorescence was measured on an Envision plate reader (Perkin Elmer) using a 485 nm excitation filter with a 14 nm bandwidth and 535 nm emission filter with a 25 nm bandwidth.

***Toxoplasma gondii* culturing and assay methods.** The tachyzoite stage of *Toxoplasma gondii* (RH1, GFP-luc) was maintained by serial passage in human foreskin fibroblasts (HFF) cultured in Hank's Buffered Salt Solution (HBSS) supplemented with 10% FBS, 2 mM L-glutamine, and 100 µg penicillin/100 µg streptomycin per milliliter maintained at 37 °C and 5% CO_2. Parasites were harvested for use in assays by syringe lysis of infected HFF monolayers. Parasites were then centrifuged at 1,200 × g for 6 min and resuspended in HBSS supplemented with 10 mM Hepes, pH 7.0, and 1% (v/v) dialyzed FBS. All buffers were filter sterilized by passage through a 5-µm Millipore Syringe Filter. Approximately 50,000 parasites and 5,000 HFF cell were incubated with compounds for 48 hours at 37 °C. Parasite and host cell proliferation rates were then determined using Bright-Glo® and CellTiter-Glo® (Promega).

***Trypanosoma brucei* culturing and assay methods.** Cells of the bloodstream form of *Trypanosoma brucei* Lister 427 were maintained as described by Hirumi and Hirumi (Hirumi and Hirumi, 1989). In brief, continuous cultures were maintained at 37 °C / 5% CO_2 by serial

passage in HMI-9 medium containing 10% FBS and 10% SerumPlus™ supplement (SAFC Biosciences). The parasite growth inhibition by cladosporin was measured after culturing *T. brucei* cells (2.5 x 10^4 cells in 50 µl) for 48 hours in the presence of different concentrations of the inhibitor. The extent of parasite growth was determined by the CellTiter-Glo® viability assay (Promega).

Leishmania donovani **culture and assay methods.** The *Leishmania donovani* 1S-Cl2D axenic amastigotes were maintained at 37 °C / 5% CO_2 by serial passage in RPMI 1640 medium supplemented with 20% heat inactivated FBS, 4 mM L-glutamine, 100 units/ml penicillin, 100 µg/ml streptomycin, 23 µM folic acid, 100 µM adenosine, 22 mM D-glucose and 25 mM 2-(N-morpholino)ethanesulfonic acid (MES), and adjusted to pH = 5.5 with HCl. To measure inhibition of amastigote growth by cladosporin, 10^4 parasite cells were grown in 50 µl of media for 48 hours in the presence of varying concentrations of the inhibitor. The extent of parasite growth was determined by the CellTiter-Glo® viability assay (Promega).

Sequencing of KRS genes from ***L. donovani*** **and** ***T. brucei.*** Genomic DNA from_*L. donovani* and *T. brucei* were isolated from parasite cells using the DNeasy Blood & Tissue Kit (Qiagen) according to the manufacturer's instructions. Fragments of each KRS gene encompassing the region encoding the ATP binding pocket were PCR-amplified from gDNA using primer sets for *L. donovani* (5'-TCACGCACCACAACGCCAACGA-3' and 5'-GATGAGCTTGTCGAACATCTTGG-3') and *T. brucei* (5'-TGAGACACTTTGTTGAGGTAGAGAC-3' and TACGGCTGGAAACTGAATGTCATG-3'). The amplicons produced by Phusion polymerase (Thermo Fisher Scientific) were cloned into pCR4Blunt-TOPO (Invitrogen) and transformed into chemically competent *E. coli* TOP10 cells.

Restriction enzyme digests with EcoRI confirmed the presence of successfully ligated *KRS* gene fragments. Direct DNA sequencing was performed on four independent clones for each construct to confirm the amino acid encoded at residues 189 and 205 for *L. donovani KRS* and residues 288 and 304 for *T. brucei KRS*.

Mammalian toxicity assays. The 50% cytoxicity inhibitory concentration (CC_{50}) of small molecule inhibitors were assessed against the following adherent, mammalian cell lines: HEp2 (human laryngeal carcinoma), HeLa (human epithelial carcinoma), HepG2 (human liver carcinoma) and Huh7 (human liver carcinoma). Cells were assayed in a 12-point dose response in 1,536-well plates seeded at a cell density of 250-750 cells per well (DMEM with 5% fetal bovine serum) 24 hours hours before compound addition. Cells were incubated in the presence of drug for 72 hours before CellTiter-Glo® reagent (Promega) was dispensed to the assay plates. Luminescence was measured on an Envision 2104 Multilabel Reader (Perkin Elmer) and dose-response signals were normalized to a DMSO-treated control and fit to a nonlinear regression curve.

Metabolic labeling with [35S]-methione and [35S]-cysteine for protein synthesis inhibition studies. Asynchronous cultures of *P. falciparum* strain Dd2 were harvested at a parasitemia of 8-10% (hematocrit of 5%), washed three times in methionine- and cysteine-free culturing media, and aliquoted in triplicate. Stock solutions were prepared with powders purchased from Sigma and dissolved in DMSO as 1000x stock solutions. All stock solutions were based on the following IC_{50} values determined by the SYBR Green-based proliferation assay: anisomycin (25 nM), cycloheximide (200 nM), artemisinin (15 nM), mefloquine (8 nM), and cladosporin (62 nM). Compounds were transferred to their respective parasite aliquots and preincubated for 15

minutes at 37°C before the addition of EasyTag™ EXPRESS ^{35}S protein labeling mix (Perkin Elmer) to a final concentration of 125 μCi/mL. Following a 1 hour pulse at 37 °C, the media was exchanged with chilled PBS supplemented with 1 mg/mL methionine and 2 mg/mL cysteine. The remaining steps were performed on ice or at 4°C when possible. The parasites were then extracted by the saponin lysis method and resuspended in PBS supplemented with 0.02% deoxycholate. TCA was immediately added to a final concentration of 8% and the resultant precipitate suspensions were transferred to a MultiScreenHTS plate with 1.2 μm FC glass and 0.65 μm Durapore membranes (Millipore). The plates were washed twice with 8% TCA and a final wash of 90% acetone. The plates were air dried at room temperature and 40 μL MicroScint-20 (Perkin Elmer) was added to each well. A TopCount microplate scintillation counter (Perkin Elmer) was used to analyze the plates. All data were normalized to TCA precipitates from untreated parasites.

Supplemental Figures

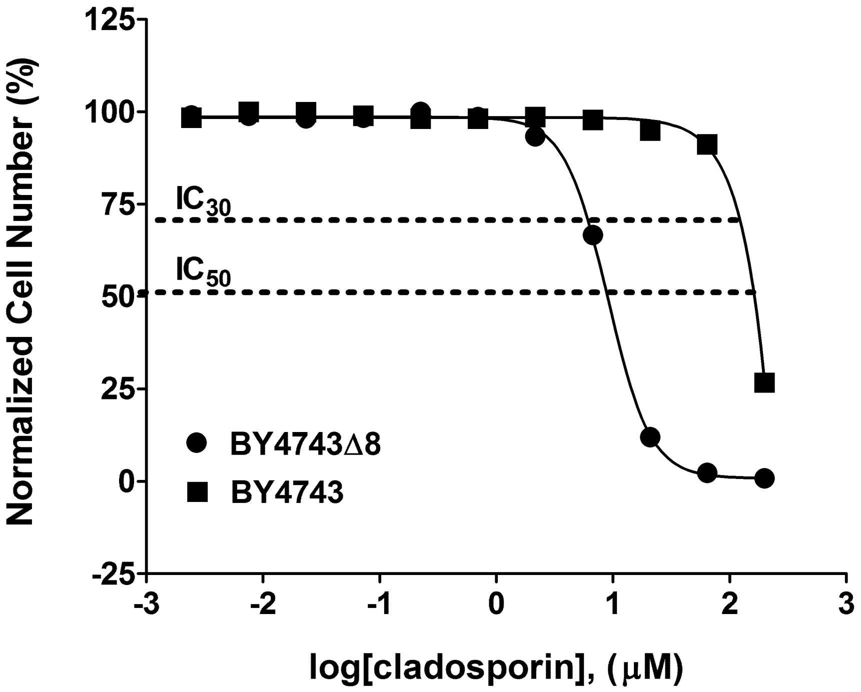

Figure S1 | Cladosporin inhibition of *S. cerevisiae* wild type strain BY4743 and a drug efflux mutant strain BY4743Δ8, related to Figure 1. Yeast growth in the presence of cladosporin was measured by optical density at 600 nm (OD_{600}) and the 30% inhibitory concentration (IC_{30}) and 50% inhibitory concentration (IC_{50}) of cell growth were determined. Assay plates seeded with the respective strains were grown in rich medium and incubated 11 hours against a 12-point dilution series of cladosporin (0-200 μM). Data were collected from duplicate experiments, normalized with a DMSO control and expressed as the means±SD. A logistic regression curve fit algorithm on the recorded OD_{600} values was performed to determine the aforementioned IC_{30} and IC_{50} values.

A

```
ScKrs1 MSQQDNVKAAAE-GVANLHLDEATGEMVSKSELKKRI------------- 36
PfKrs1 MTSKSFLLSFLKYKHVNTYIFEKSFSKILKNT-KKHIDCHLKSCFVTMNE 49
       *:.:. : :  :   .* :: * : . : *.  **:*

ScKrs1 -KQRQVEAKKAAKKAAAQPKPASKKKTDLFADLDPSQYFETRSRQIQELR 85
PfKrs1 KKEHVLEGEKNKRVVN----ASKDKKKEEEGEVDPRLYFENRSKFIQD-Q 94
       *:: :*.:*  : .     .:..**.:  .::**  ***.**: **: :

ScKrs1 KTHEPNPYPHKFHVSISNPEFLAKYAHLKKGETLPEEKVSIAGRIHAKRE 135
PfKrs1 KDKGINPYPHKFERTISIPEFIEKYKDLGNGEHLEDTILNITGRIMRVSA 144
       * :  *******. :** ***: ** .* :** * :  :.*:***

ScKrs1 SGSKLKFYVLHGDGVEVQLMSQLQDY-CDPDSYEKDHDLLKRGDIVGVEG 184
PfKrs1 SGQKLRFFDLVGDGEKIQVLANYSFHNHEKGNFAECYDKIRRGDIVGIVG 194
       **.**:*: * *** ::*:::: . :  : ..: : :* ::******: *

ScKrs1 YVGRTQPKKGGEGEVSVFVSRVQLLTPCLHMLPADHFGFKDQETRYRKRY 234
PfKrs1 FPGKS-----KKGELSIFPKETILLSACLHMLPMK-YGLKDTEIRYRQRY 238
       : *::      :**:*:* ... **:.****** . :*:** * ***:**

ScKrs1 LDLIMNKDARNRFITRSEIIRYIRRFLDQRKFIEVETPMMNVIAGGATAK 284
PfKrs1 LDLLINESSRHTFVTRTKIINFLRNFLNERGFFEVETPMMNLIAGGANAR 288
       ***::*:.:*: *:**::**.::*.**::* *:********:*****.*:

ScKrs1 PFITHHNDLDMDMYMRIAPELFLKQLVVGGLDRVYEIGRQFRNEGIDMTH 334
PfKrs1 PFITHHNDLDLDLYLRIATELPLKMLIVGGIDKVYEIGKVFRNEGIDNTH 338
       **********:*:*:***.** ** *:***:*:*****: ******* **

ScKrs1 NPEFTTCEFYQAYADVYDLMDMTELMFSEMVKEITGSYIIKYHPDPADPA 384
PfKrs1 NPEFTSCEFYWAYADYNDLIKWSEDFFSQLVYHLFGTYKISYNKDGPE-N 387
       *****:**** ****  **:. :* :**::* .: *:* *.*: * .:
     T340I

ScKrs1 KELELNFSRPWKRINMIEELEKVFNVKFPSGDQLHTAETGEFLKKILVDN 434
PfKrs1 QPIEIDFTPPYPKVSIVEEIEKVTNTILEQ--PFDSNETIEKMINIIKEH 435
       : :*::*: *: ::.::**:*** *. : .   :.: ** * : :*: ::

ScKrs1 KLECPPPLTNARMLDKLVGELEDTCIN--PTFIFGHPQMMSPLAKYSRDQ 482
PfKrs1 KIELPNPPTAAKLLDQLASHFIENKYNDKPFFIVEHPQIMSPLAKYHRTK 485
       *:* * * * *::**:*...: :.  *  * **. ***:******* * :

ScKrs1 PGLCERFEVFVATKEICNAYTELNDPFDQRARFEEQARQKDQGDDEAQLV 532
PfKrs1 PGLTERLEMFICGKEVLNAYTELNDPFKQKECFKLQQKDREKGDTEAAQL 535
       *** **:*:*:. **: **********.*:  *: * :::::** **  :

ScKrs1 DETFCNALEYGLPPTGGWGCGIDRLAMFLTDSNTIREVLLFPTLKPDVLR 582
PfKrs1 DSAFCTSLEYGLPPTGGLGLGIDRITMFLTNKNSIKDVILFPTMRP---- 581
       *.:**.:*********** * ****::****:.*:*::*:****::*
                    G551S             I567V

ScKrs1 EEVKKEEEN 591
PfKrs1 -------AN 583
               *
```

B

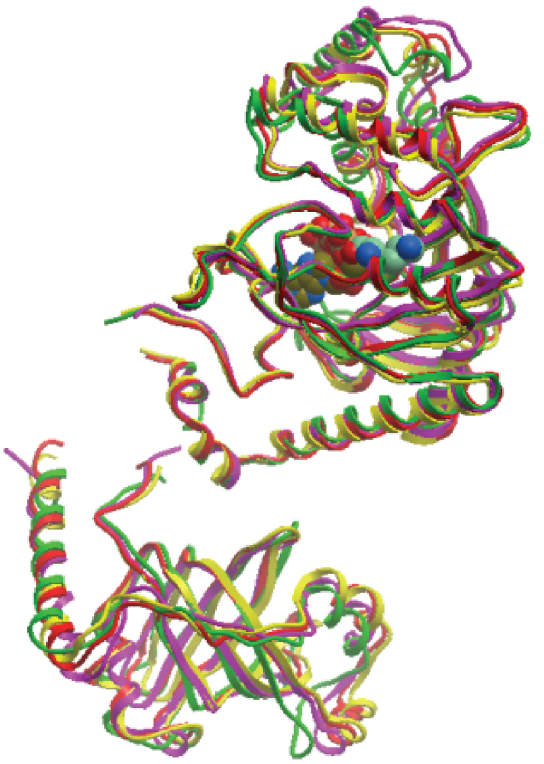

Figure S2 | Amino acid and structural similarities amongst Krs1 from different species, related to Figure 2. (A) Pairwise alignment of the primary sequences of Krs1 (GenBank accession: CAA98863.1) and the *Plasmodium falciparum* homolog (PF13_0262) using ClustalW2 (version 2.1). The alignment scored 44.2 % identity and 58.5 % similarity between amino acid residues. Resistance-conferring mutations identified by direct DNA sequencing of cladosporin-resistant yeast strains are labeled. (B) Superimposition of the human lysyl-tRNA synthetase and the bacterial X-ray crystal structures. Ribbon representation of the human lysyl-tRNA synthetase structure (PDB ID: 3BJU) is shown in green, the *Escherichia coli* structure (PDB ID: 1BBU; r.m.s.d. = 1.81 Å with 3BJU) in orange, and the *Bacillus stearothermophilus* structure (PDB ID: 3E9H; r.m.s.d. = 1.89 Å with 3BJU) in magenta. The ATP and lysine structures of the human protein are shown as space-filling models (brown and cyan, respectively).

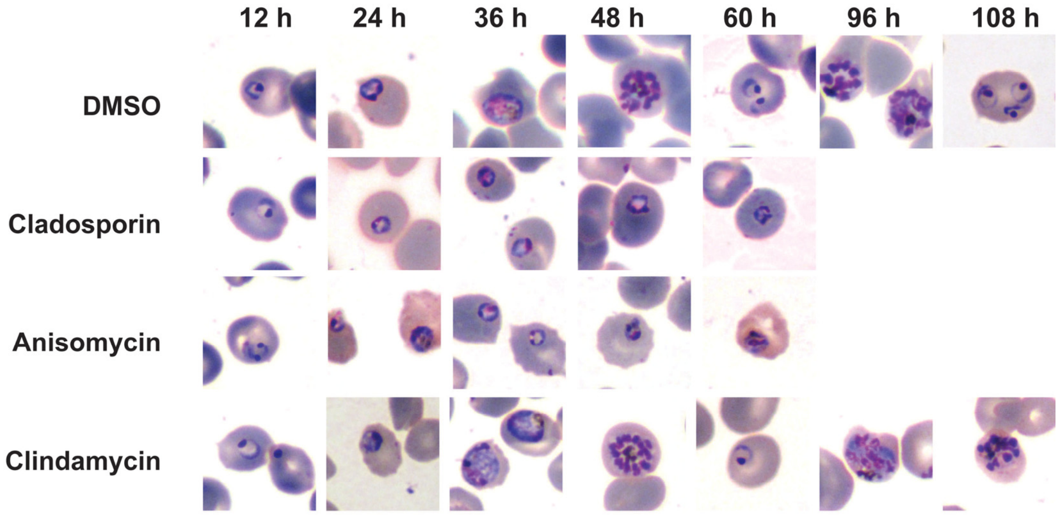

Figure S3 | Stage of action of cladosporin, anisomycin and clindamycin in synchronized *P. falciparum* parasites, related to Figure 3C. Cladosporin's mode of action was evaluated against synchronized blood-stage parasites to distinguish whether cladosporin was fast-acting (onset of action in the first generation) or induced a delayed death phenotype (onset of action in the second generation). The morphology of parasites was monitored by Giemsa-stained thin blood smears over a 60 or 108-hour period (1.25 or 2.25 life cycles). Untreated parasites (0.1% DMSO) were used as a control and the following drugs were administered at 10x their IC_{50} value: 400 nM cladosporin, 250 nM anisomycin, or 110 nM clindamycin. Representative images are shown for each time point.

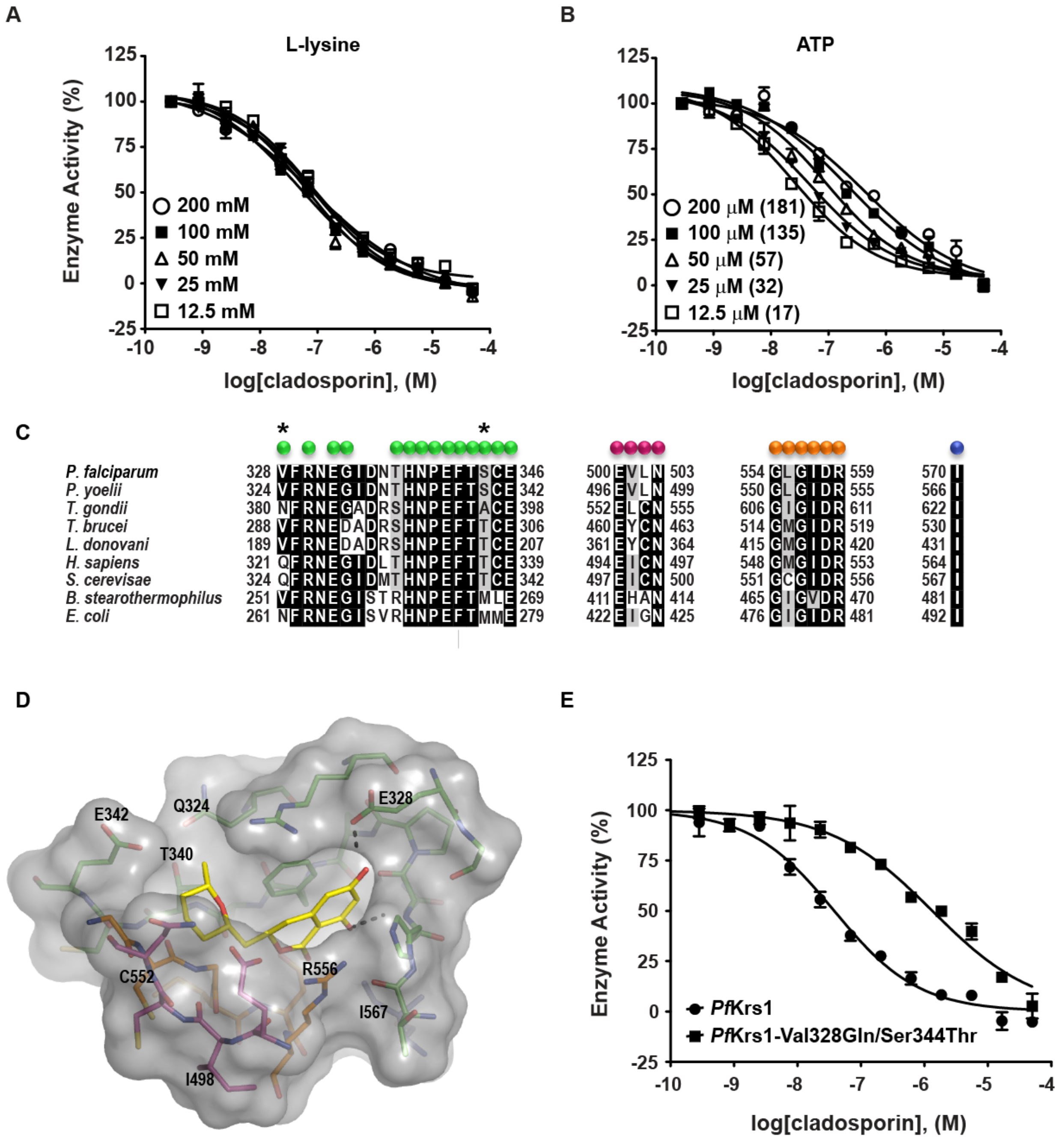
A
L-lysine
Enzyme Activity (%)
log[cladosporin], (M)
200 mM
100 mM
50 mM
25 mM
12.5 mM
B
ATP
200 μM (181)
100 μM (135)
50 μM (57)
25 μM (32)
12.5 μM (17)
log[cladosporin], (M)
C
P. falciparum
P. yoelii
T. gondii
T. brucei
L. donovani
H. sapiens
S. cerevisae
B. stearothermophilus
E. coli
D
E342
Q324
E328
T340
C552
R556
I567
I498
E
Enzyme Activity (%)
PfKrs1
PfKrs1-Val328Gln/Ser344Thr
log[cladosporin], (M)

Figure S4 | Cladosporin binds the ATP pocket of Krs1 where two amino acids dictate selectivity, related to Figure 4. Recombinant *Pf*Krs1 was assayed in the presence of increasing (A) lysine and (B) ATP concentrations in the standard reaction buffer. The IC_{50} of cladosporin remained unaffected from lysine concentrations between 0-200 mM. However, incremental, two-fold increases of ATP in the assay buffer resulted in decreased cladosporin inhibition. The corresponding cladosporin IC_{50} value (expressed as μM) is given in parentheses next to the ATP concentration in the symbol legend. These data are expressed as the means±SEM of two independent experiments run in triplicate. A logistic regression curve fit algorithm on the normalized enzymatic activity was performed to determine the IC_{50} value. (C) ClustalW2 amino acid alignment of the ATP binding site residues between species with known cladosporin activity (Table 2). Positions of the key residues that contribute to cladosporin selectivity are starred. Color-coded dots above the alignment correspond to the color-coded residues shown in panel D. (D) Stick representation of cladosporin (yellow) docked to a homology model of yeast lysyl-tRNA synthetase. Yeast amino acid residues are also shown in stick representation with a transparent overlay of the corresponding Van der Waal's surface area (grey). Specific residues are labeled for clarity. (E) "Yeastification" of amino acid residues 328 and 344 in the ATP-binding site of *Pf*Krs1 confers cladosporin resistance. Recombinant wild type *Pf*Krs1 and a double mutant with conservative changes to the corresponding yeast residue (Val328Gln and Ser344Thr) were assayed for cladosporin sensitivity. The double mutations conferred significant resistance to cladosporin (IC_{50} = 983 nM; 32.8-fold change). The fold change in cladosporin potency was remarkably similar to that in "plasmodified" *Sc*Krs1 double mutant expressed in yeast (Figure 4D; induced a 38.7-fold change). These data are expressed as the means±SEM of two independent experiments run in triplicate. A logistic regression curve fit algorithm on the normalized enzymatic activity was performed to determine the IC_{50} value for each.

SUPPLEMENTAL TABLES

Table S1. Quantification of cidal and static properties of cladosporin against malaria parasites, related to Table 1.

	Timing in parasite lifecycle (h)			
**line**	**44**	**68**	**92**	**112**
**untreated Dd2**	2.7% (mid-schizont)	7.2% rings	7.4% (mid-schizont)	>11% rings
**drug-treated Dd2**	2.7% (early trophozoite)	1.3% (late trophozoite)	1.0% (schizont)	0.7% rings

Dd2 parasites were synchronized to within a 2-hour window using magnetic microbeads (MACS, Miltenyi Biotec) and resuspended at ~2.7% parasitemia before cladosporin addition (400 nM, 10x IC_{50}). After 44 hours (mid-schizogony, before egress), cladosporin was rinsed from the cells (3 washes x 5 ml culturing media). Then the parasites were continually monitored for another two egress/reinvasion cycles. Parasitemia was determined at the indicated times. The major parasite form is given in parentheses.

Table S2. Potency of cladosporin on yeast strains in variant growth conditions and experiments, related to Figure 1.

				Cladosporin				
**Strain description**	**Strain ploidy**	***KRS1* copy number/ ploidy ratio**	**Medium**	**IC_{30} [μM]**	**IC_{50} [μM]**	**MIC [μM]**	**IC_{50} shift vs control**	**Experiment**
BY4743	2n	1	Rich, Glucose	65-120[1]	93-200[1]	50		HIP HOP
BY4743Δ8 (deleted for 8 drug resistance genes)	2n	1	Rich, Glucose	5.3	7.7			Mutagenesis
BY4741 plasmid-encoded *S.c. GLN4* under GAL control	1n	1	Synthetic, -Uracil, Galactose		58.9		control	Synthetase overexpression
BY4741 plasmid-encoded *S.c. ILS1* under GAL control	1n	1	Synthetic, -Uracil, Galactose		51.4		control	Synthetase overexpression
BY4741 plasmid-encoded *S.c. THS1* under GAL control	1n	1	Synthetic, -Uracil, Galactose		51.0		control	Synthetase overexpression
BY4741 plasmid-encoded *S.c. KRS1* under GAL control	1n	2	Synthetic, -Uracil, Galactose		112.2		2.0x	Synthetase overexpression
BY4741Δ8 empty vector	1n	1	Synthetic, -Uracil, Glucose		26.7		control	Mutagenesis validation
BY4743Δ8 plasmid-encoded *S.c. KRS1*	1n	2	Synthetic, -Uracil, Glucose		89.0		3.3x	Mutagenesis validation
BY4741Δ8 plasmid-encoded *S.c. KRS1Ile567Val*	1n	2	Synthetic, -Uracil, Glucose		143.2		5.4x	Mutagenesis validation
BY4741Δ8 plasmid-encoded *S.c. KRS1Gly551Ser*	1n	2	Synthetic, -Uracil, Glucose		142.4		5.3x	Mutagenesis validation
BY4741Δ8 plasmid-encoded *S.c. KRS1Thr340Ile*	1n	2	Synthetic, -Uracil, Glucose		139.9		5.2x	Mutagenesis validation
BY4741 *krs1Δ* plasmid-encoded *S.c. KRS1*	1n	1	Synthetic, -Uracil, Glucose		162.6		control	Specificity sites
BY4741 *krs1Δ* plasmid-encoded *S.c. KRS1 Gln324Val*	1n	1	Synthetic, -Uracil, Glucose		28.3		-5.7x	Specificity sites
BY4741 *krs1Δ* plasmid-encoded *S.c. KRS1 Thr340Ser*	1n	1	Synthetic, -Uracil, Glucose		15.5		-10.4x	Specificity sites
BY4741 *krs1Δ* plasmid-encoded *S.c. KRS1 Gln324Val / Thr340Ser*	1n	1	Synthetic, -Uracil, Glucose		4.2		-38.7x	Specificity sites
BY4741 *krs1Δ* plasmid-encoded *S.c. KRS1*	1n	1	Synthetic, -Uracil, Glucose		>200		control	Heterologous synthetases
BY4741 *krs1Δ* plasmid-encoded, hybrid *S.c./H.s. KRS1*	1n	1	Synthetic, -Uracil, Glucose		>200		~1.0x	Heterologous synthetases
BY4741 *krs1Δ* plasmid-encoded, hybrid *S.c./P.f. KRS1*	1n	1	Synthetic, -Uracil, Glucose		0.2		>1000x	Heterologous synthetases
BY4743Δ8	2n	1	Rich, Glucose		7.9		control	Lysine competition
BY4743Δ8	2n	1	Rich, Glucose, +32 mM Lys		7.9		1.00x	Lysine competition
BY4743Δ8	2n	1	Synthetic, Glucose		18.4		control	Lysine competition
BY4743Δ8	2n	1	Synthetic, Glucose, +32 mM Lys		18.9		1.03x	Lysine competition

[1]Different compound batches were used for experiment repeats

Table S3. Summary of genome scanning and IC_{50} values in drug-evolved and parental lines, related to Figure 3A.

Line	Genomic changes		IC_{50} (nM)[3]
	CNV[1]	SNP[2]	cladosporin
**Dd2 parent**	none	--	62.5 ± 3.5
**cladosporin-R^{Dd2} clone#1 (R1)**	amplification Chr13	none	377.1 ± 31.4
**cladosporin-R^{Dd2} clone#2 (R2)**	amplification Chr13	two[4]	389.5 ± 39.6
**cladosporin-R^{Dd2} clone#3 (R3)**	amplification Chr13	none	374.7 ± 54.0

[1]CNV: copy number variation

[2]SNP: single nucleotide polymorphism; SNPs detected below the cut-off ($P < 10^{-5}$) and/or reside in genes involved in antigenic variation (rifin and pfemp1) were excluded.

[3]IC_{50} values are represented as means±SD and were calculated from two independent experiments performed in duplicate measurements with the SYBR Green cell proliferation assay.

[4]likely SNP in MAL13P1.310 (calpain) and PF14_0501 (conserved *Plasmodium* protein).

Table S4. IC_{50} values of cladosporin-R^{Dd2} clones against a panel of antimalarials with unique mechanism of action[1], related to Figure 3A.

Line	IC_{50} values (nM)[2]						
	ANI	ART	ATQ	CycA	MFQ	NITD678	PYR
**Dd2**	19.5 ± 1.0	17.0 ± 1.2	0.8 ± 0.2	48.8 ± 10.1	9.0 ± 0.9	21.7 ± 1.3	>10,000
**R1**	17.9 ± 2.2	14.9 ± 1.1	1.1 ± 0.3	32.4 ± 8.7	7.8 ± 1.4	24.0 ± 7.6	>10,000
**R2**	17.6 ± 1.5	18.7 ± 3.1	0.5 ± 0.1	60.9 ± 12.6	11.3± 2.3	26.4 ± 5.7	>10,000
**R3**	19.7 ± 2.4	15.6 ± 1.6	0.6 ± 0.1	45.5 ± 7.4	7.5 ± 1.1	18.5 ± 2.2	>10,000

[1]abbreviations: anisomycin (ANI), artemisinin (ART), atovaquone (ATQ), cyclomarin A (cycA), mefloquine (MFQ), a less potent derivative of NITD609 (NITD678), and pyrimethamine (PYR).
[2]IC_{50} values are represented as means±SD and were calculated from two independent experiments performed in duplicate with the SYBR Green cell proliferation assay.

Table S5. Exogenous lysine in growth media does not alter cladosporin activity in yeast, related to Figure 4.

	Cladosporin IC_{50} values (μM)[1]	
**[Lysine], mM**	**Synthetic media**	**Rich media**
**0.0**	18.42	7.92
**1.08**	18.30	8.26
**3.36**	19.28	8.41
**10.41**	19.65	7.99
**32.26**	18.94	7.94
**100.00**	15.95	7.02

[1]IC_{50} values are represented as the mean calculated from two independent experiments performed in duplicate measurements with the optical density assay against yeast line BY4743Δ8.

SUPPLEMENTAL REFERENCES

Dharia, N.V., Sidhu, A.B., Cassera, M.B., Westenberger, S.J., Bopp, S.E., Eastman, R.T., Plouffe, D., Batalov, S., Park, D.J., Volkman, S.K., *et al.* (2009). Use of high-density tiling microarrays to identify mutations globally and elucidate mechanisms of drug resistance in Plasmodium falciparum. Genome Biol *10*, R21.

Hirumi, H., and Hirumi, K. (1989). Continuous cultivation of Trypanosoma brucei blood stream forms in a medium containing a low concentration of serum protein without feeder cell layers. J Parasitol *75*, 985-989.

Lambros, C., and Vanderberg, J.P. (1979). Synchronization of Plasmodium falciparum erythrocytic stages in culture. J Parasitol *65*, 418-420.

Meister, S., Plouffe, D., Kuhen, K., Bonamy, G., Barnes, S., Bopp, S., Borboa, R., Bright, A., Che, J., Cohen, S., *et al.* (2011). Exploring Plasmodium hepatic stages to find next-generation antimalarial drugs. Science *334*, 1372-1377.

Plouffe, D., Brinker, A., McNamara, C., Henson, K., Kato, N., Kuhen, K., Nagle, A., Adrian, F., Matzen, J.T., Anderson, P., *et al.* (2008). In silico activity profiling reveals the mechanism of action of antimalarials discovered in a high-throughput screen. Proc Natl Acad Sci U S A *105*, 9059-9064.

Rénia, L., Mattei, D., Goma, J., Pied, S., Dubois, P., Miltgen, F., Nüssler, A., Matile, H., Menégaux, F., Gentilini, M., *et al.* (1990). A malaria heat-shock-like determinant expressed on the infected hepatocyte surface is the target of antibody-dependent cell-mediated cytotoxic mechanisms by nonparenchymal liver cells. European Journal of Immunology *20*, 1445-1449.

Yalaoui, S., Zougbede, S., Charrin, S., Silvie, O., Arduise, C., Farhati, K., Boucheix, C., Mazier, D., Rubinstein, E., and Froissard, P. (2008). Hepatocyte permissiveness to Plasmodium infection is conveyed by a short and structurally conserved region of the CD81 large extracellular domain. PLoS Pathog *4*, e1000010.
